# C-952-02. Assessing Orbital Compartment Syndrome Management in the Burns ICU: A Retrospective Chart Review

**DOI:** 10.1093/jbcr/irag033.118

**Published:** 2026-04-06

**Authors:** Abdullahi A Mohamed, Justin J Lee, Matthew D Benson, Joshua N Wong

**Affiliations:** University of Alberta, Edmonton, AB; University of Alberta, Edmonton, AB; University of Alberta, Edmonton, AB; University of Alberta, Edmonton, AB

## Abstract

**Introduction:**

Orbital compartment syndrome (OCS) is a vision-threatening emergency. In burn patients, it remains understudied, without standardized screening guidelines. This study assesses OCS incidence, risk factors, outcomes, and evaluates proposed guidelines in a burns ICU setting.

**Methods:**

A retrospective observational cohort study was conducted on patients with ≥20% total body surface area (TBSA) burns admitted to a burn ICU from January 2022 to January 2024 (n = 64). Data collected included demographics, TBSA, burn distribution, escharotomy frequency, 24-hour fluid resuscitation volumes, incidence of OCS, and lateral canthotomy and cantholysis (LCC). Pre- and post-LCC intraocular pressure (IOP) and timing of OCS diagnosis, visual outcomes at ICU discharge, and complications related to OCS or LCC were also reviewed. Proposed screening criteria (TBSA >40%, facial burns, or resuscitation >80 mL/kg) were retrospectively applied to assess diagnostic performance. Comparative analyses between OCS and non-OCS patients were performed using independent t-tests.

**Results:**

Seven (10.9%) developed OCS, all with facial burns and multiple anatomical regions involved, requiring bilateral LCC. The mean time to LCC was 4.7 hours. Compared with non-OCS patients, those with OCS had larger burns (mean TBSA 49%), higher 24-hour fluid volumes (14.6 L), and more escharotomies (5.1) (*p*<.05). Mean IOP decreased from 38.7 to 26.2 mmHg after LCC. Retrospective application of proposed screening criteria (TBSA >40%, facial burns, or resuscitation >80 mL/kg) identified all OCS cases, yielding sensitivity of 100% and specificity of 49%.

**Conclusions:**

This study provides one of the largest single-center evaluations of OCS in the burn ICU setting. Large TBSA, facial burns, high resuscitation volumes, and multiple escharotomies emerged as key risk factors. Proposed screening criteria demonstrated excellent sensitivity, supporting their potential role in earlier detection and timely LCC. These findings underscore the need for prospective validation and standardized protocols to reduce preventable vision loss.

**Applicability of Research to Practice:**

By identifying high-risk features and demonstrating the diagnostic utility of preliminary screening criteria, this study highlights a feasible pathway for earlier OCS recognition in burn patients. Adoption of structured screening could improve detection, reduce delays in intervention, enhance visual outcomes, and guide the development of standardized OCS screening guidelines. The integration of guideline development and diagnostic testing represents a novel approach to addressing a critical gap in ocular and burn emergency care by providing a framework for OCS screening in the burn ICU setting.

**Funding for the study:**

Foundation Funding for Summer Research Scholarship from the Faculty of Medicine & Dentistry.

